# Policy implementation strategies to address rural disparities in access to care for stroke patients

**DOI:** 10.3389/frhs.2023.1280250

**Published:** 2023-11-30

**Authors:** Aysha Rasool, Moriah Bailey, Brittany Lue, Nina Omeaku, Adebola Popoola, Sharada S. Shantharam, Amanda A. Brown, Erika B. Fulmer

**Affiliations:** ^1^ Division for Heart Disease and Stroke Prevention, Centers for Disease Control and Prevention, Atlanta, GA, United States; ^2^ Oak Ridge Institute for Science and Education, Oak Ridge, TN, United States; ^3^ Applied Science, Research and Technology, Inc., Atlanta, GA, United States; ^4^ Chenega Corporation, Anchorage, AK, United States

**Keywords:** stroke, policy, emergency medical services (EMS), rural, disparity

## Abstract

**Context:**

Stroke systems of care (SSOC) promote access to stroke prevention, treatment, and rehabilitation and ensure patients receive evidence-based treatment. Stroke patients living in rural areas have disproportionately less access to emergency medical services (EMS). In the United States, rural counties have a 30% higher stroke mortality rate compared to urban counties. Many states have SSOC laws supported by evidence; however, there are knowledge gaps in how states implement these state laws to strengthen SSOC.

**Objective:**

This study identifies strategies and potential challenges to implementing state policy interventions that require or encourage evidence-supported pre-hospital interventions for stroke pre-notification, triage and transport, and inter-facility transfer of patients to the most appropriate stroke facility.

**Design:**

Researchers interviewed representatives engaged in implementing SSOC across six states. Informants (*n* = 34) included state public health agency staff and other public health and clinical practitioners.

**Outcomes:**

This study examined implementation of pre-hospital SSOCs policies in terms of (1) development roles, processes, facilitators, and barriers; (2) implementation partners, challenges, and solutions; (3) EMS system structure, protocols, communication, and supervision; and (4) program improvement, outcomes, and sustainability.

**Results:**

Challenges included unequal resource allocation and EMS and hospital services coverage, particularly in rural settings, lack of stroke registry usage, insufficient technologies, inconsistent use of standardized tools and protocols, collaboration gaps across SSOC, and lack of EMS stroke training. Strategies included addressing scarce resources, services, and facilities; disseminating, training on, and implementing standardized statewide SSOC protocols and tools; and utilizing SSOC quality and performance improvement systems and approaches.

**Conclusions:**

This paper identifies several strategies that can be incorporated to enhance the implementation of evidence-based stroke policies to improve access to timely stroke care for all patient populations, particularly those experiencing disparities in rural communities.

## Introduction

Individuals living in rural areas are more likely to have limited access to health care, are less likely to be insured, and are more likely to live in poverty (1). Stroke patients living in rural areas experience worse health outcomes and 30% higher in-patient mortality rates when compared to stroke patients living in urban areas (2). Existing disparities in stroke incidence, health care infrastructure, and the geographic maldistribution of services contribute to widening disparities in stroke care outcomes and access to appropriate, timely treatment for historically underserved populations (3–5). Rapid transport to hospitals capable of administering thrombolytic agents and endovascular treatments within a few hours of stroke onset are essential components in the treatment of ischemic strokes, the most common type of stroke (6).

The adoption of stroke center certification is associated with patients' access to lifesaving stroke treatment (4), and while inequalities in access to certified stroke centers in rural areas have lessened in recent years, differences in mortality outcomes and the use of alteplase and thrombectomy treatments have widened (7). These variations in mortality outcomes may be due to a lack of timely evaluation and treatment in rural communities, a slower rate of growth in the use of thrombectomy and alteplase, lack of access to neurology input, and differences in social determinants of health, such as income and education (8).

**Figure 1:**
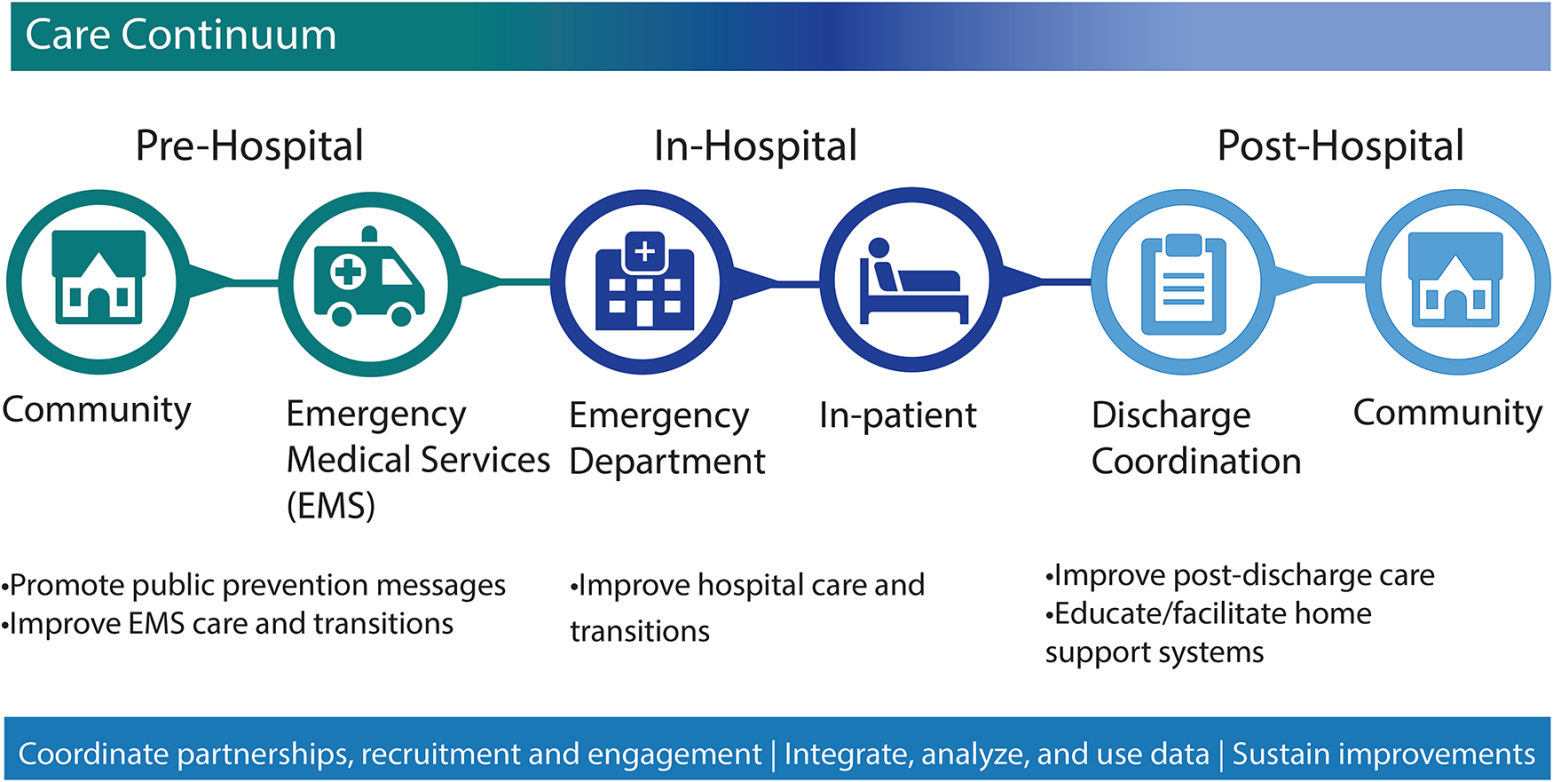
Stroke systems of care continuum. CDC. About the Paul Coverdell National Acute Stroke Program. www.cdc.gov. Published November 28, 2018. Accessed November 10, 2022. Available at: https://archive.cdc.gov/#/details?url=https://www.cdc.gov/dhdsp/programs/about_pcnasp.htm. ^1^EMS, emergency medical services.

We identified potential strategies and challenges within several states in implementing state policy interventions^1^ that require or otherwise encourage evidence-supported pre-hospital interventions for stroke pre-notification, triage and transport, and inter-facility transfer of patients to the most appropriate stroke facility. Additionally, the paper considers implementation strategies and challenges unique to rural populations seeking to strengthen the coordination of services within their pre-hospital care setting (See Figure 1). These findings may support state health departments, policymakers, Emergency Medical Services (EMS) Agencies, EMS coalitions, hospitals, and stroke coordinators when implementing SSOC policies in the pre-hospital setting in both rural and urban settings.

## Methods

We conducted a case study, selecting states as the primary unit of analysis. Five selection criteria were applied to ensure that included states had robust yet varied SSOC laws and were geographically diverse. These criteria included: (1) the number and type of evidence-informed pre-hospital policy interventions addressed in the state law; (2) whether state SSOC policy was legislative, regulatory, or both; (3) whether the state has a state level, regional or local policy approach to pre-hospital care; (4) home rule status^2^; and (5) the extent to which the state is urban vs. rural. The total number of included states was constrained by available resources and the desire to capture a broader array of perspectives within each selected state. Table 1 lists how each case study state met the selection criteria and the percentage of states not included in the case study that also met the selection criteria. Seven out of 45 (15.5%) remaining states and D.C. that were not included in the case study met one or more of the inclusion criteria.

**Table 1:** Characteristics of case study states.

Characteristic of states	GA	IL	LA	MO	RI	WY	States not selected for case study that met criteria (%)
Authorization of statewide and/or regional EMS protocols for stroke patient assessment and triage^a^	X	X	X	X	X	X	15/45 (33.3)
Authorization of statewide and/or regional EMS transport protocols for transporting the stroke patient to the most appropriate stroke facility using ground transport	X	X	X	X	X	X	–
Authorization of inter-facility transfer agreements to transfer the stroke patient to the most appropriate stroke facility	X	X	X	X	X	X	–
Authorization of stroke pre-notification by EMS to the receiving facility			X	X		X	–
Does/did the state have a state-level stroke-specific task force or advisory committee?	X	X	X	X	X		41/45 (91.1)
Does the state require stroke facilities/EMS to report data on outcomes to a state-level registry^a^?	X			X	X		4/45 (8.8)
Does state law expressly authorize a statewide/regional stroke system(s) of care with at least 3 levels of stroke centers^a^?	X	X	X	X	X	X	15/45 (33.3)
Is this a Home Rule state^a,b^?	X	X	X	X	X	X	7/45 (15.5)
State(s) have both legislative^c^ and regulatory^d^ approaches that *expressly required* one or more agencies to promulgate rules for the stroke system of care and these states have at least 3 levels of stroke centers and expressly address multiple pre-hospital stroke EMS policies^a^				X		X	–
State(s) have both legislative and regulatory approaches where the legislation *did not expressly require* one or more agencies to promulgate rules for the stroke system of care and these states have at least 3 levels of stroke centers and expressly address multiple pre-hospital stroke EMS policies^a^	X	X					–
State(s) have a legislative approach that do not require agency rule-making^e^ and to date no stroke specific rule-making has occurred^a^					X		–
State(s) have a regulatory approach and these states have at least 3 levels of stroke centers and expressly address multiple pre-hospital stroke EMS policies^a^			X				–
Majority of state population residing in nonmetropolitan category^a^	X	X	X	X		X	–
State(s) policy included a task force^a^	X	X	X		X	X	–

GA, Georgia; IL, Illinois; LA, Louisiana; MO, Missouri; RI, Rhode Island; WY, Wyoming; EMS, emergency medical service.

Barbero C, Bhuiya A, Shantharam S, Taylor L, Fulmer E, Chowdhury FM, et al. *What is the evidence for state laws to enhance in-hospital and post-hospital stroke care?* Atlanta, GA: Centers for Disease Control and Prevention, US Dept of Health and Human Services (2018).

Source for legislative: Cornell Law School. Legislation. LII/Legal Information Institute. Published 2019. Accessed November 3, 2022. Available at: https://www.law.cornell.edu/wex/legislation.

Source for metropolitan category: Ingram DD, Franco SF. 2013 NCHS urban-rural classification scheme for counties. National Center for Health Statistics. Vital Health Stat 2(166). (2014).

Source for rulemaking: Office of the Federal Register. A guide to the rulemaking process. (2010). Accessed November 3, 2022. Available at: https://www.federalregister.gov/uploads/2011/01/the_rulemaking_process.pdf.

^a^ Inclusion criteria, applied as of January 9th, 2019.

^b^ Home rule status refers to the authority delegated to local governments to enact their own laws and policies to address issues of local concern.

^c^ Legislative refers to the preparation and enactment of laws by a legislative body through its lawmaking process.

^d^ Regulatory approaches assume states are acting under a broader authority to promulgate rules.

^e^ Rulemaking agencies get their authority to issue regulations from laws (statutes) enacted by Congress.

Six states (Georgia, Illinois, Louisiana, Missouri, Rhode Island, and Wyoming) were selected for inclusion. The state EMS/Medical Director in each case study state was contacted to help determine appropriate state interview informants from state health departments and their delegates. To aid representation across states and variation in the interviewees' perspectives, several “delegate categories” were determined. Initial contacts were asked to recruit up to six stakeholders from the following eight delegate categories: State Health Agency; Disability; Rural; Data; EMS/Ambulatory; Hospital/Hospital Association; Home Health; State Associations/Taskforce. In Coverdell-funded states, CDC worked with both the Coverdell program coordinator and state EMS/Medical Director to identify informants. Out of a total of 34 informants, roles included 17 state public health staff, including public health epidemiologists and EMS/ambulatory representatives (state EMS medical directors, state EMS task force leaders, EMS training directors, and state EMS data managers) as well as 17 other public health and clinical practitioners, including stroke program coordinators, regulatory affairs staff, hospital and rural health association staff, neurologists, emergency department physicians, registered nurses, and time critical diagnosis unit managers. Additionally, one informant represented a national health-oriented nonprofit organization.

The interview guide, developed from subject matter expert input and from existing policy evidence research (10), included 29 open-ended questions that covered the following topics related to policy implementation to enhance pre-hospital SSOC:

• Development roles, processes, facilitators, and barriers.
• Implementation stakeholders, challenges, and potential solutions.
• EMS system structure, protocols, communication, and supervision.
• Program improvement, outcomes, and sustainability of state-level interventions.

A CDC human subjects review determined that the evaluation did not constitute human subjects research and, therefore, did not require IRB. Researchers completed informant interviews between August and October 2019. All interviews were conducted by telephone and lasted approximately 90 min. Some interviews were attended by more than one informant. Each was audio-recorded and professionally transcribed through a third-party service. Interviewers took notes during conversations and used the notes to verify the accuracy and completeness of the transcriptions. All data, including specific informant titles, were deidentified.

## Data analysis

Inductive thematic analysis techniques described by Green and colleagues were used to analyze the transcriptions (11). An initial codebook was developed based on subject matter expert input, topics included in the interview guide, and a thorough review of interview transcripts. Seven coders independently reviewed and analyzed the transcripts using Dedoose software version 9.0.46 (12). Coders were assigned to pairs and met to review all coding decisions and reconcile any discrepancies. If pairs could not reconcile differences, the decision was brought to a third coder to make the final determination on the appropriate code. Additional codes were discussed by the project team and added if appropriate. Once all coding was complete, individual parent and child codes were mapped to 18 high-level themes from the interview guide. These high-level themes included development and implementation processes of policies, relevant roles/key players, as well as barriers and facilitators. Eighteen datasets from coded data were created in Dedoose and downloaded in individual Excel files for further analysis. Project team members, again in pairs, were assigned to one or more high-level themes and reviewed the data. Major themes and subthemes were noted in each table, along with exemplary quotes, state, transcript number, and notes. Two authors reviewed each thematic table to distill the most salient themes that aligned with the scope of the study, aim of the anticipated publication, and relevancy to their organization's audiences. The most salient themes were compiled in a Word document, divided by sub-headers, with quotations. The number of times a sentiment was repeated by more than one respondent was noted; similarly, contradicting sentiments from respondents were also noted. A series of meetings to craft organization of findings through the most salient themes were used to finalize findings.

## Results

Results are organized by three broad categories of challenges, each followed by related strategies. The first category pertains to the coverage of pre-hospital services, the lack of resources, and the availability of facilities. The second category focuses on statewide SSOC protocols. The third category pertains to SSOC quality and performance improvement. An emerging theme we identified was the exacerbation of policy implementation challenges experienced in rural settings. Although not the primary focus of the initial study, it was an important theme and is the focus of this paper. Additional key themes are found in Table 2 and supporting quotations from the interviews are in Supplemental Digital Content Supplementary Table S1.

**Table 2:** Key themes.

Interview questions	Key themes
• Are there regional differences in the rollout?	• Representation and use of advisory boards
• How do state agencies work with EMS agencies and clinical settings to support the development process?	
• What are the greatest obstacles for pre-hospital stroke treatment in your state?	• Feedback from hospitals to EMS providers • Importance of having a protocol in place • Continuing education of EMT personnel
• What is the easiest part of the development process?	
• What aspects of healthcare culture promote (or hinder) pre-hospital stroke care?	
• What are the greatest obstacles for pre-hospital stroke treatment in your state?	• Inconsistency with EMS personnel availability • Challenges with EMS personnel following protocol • Relationship between hospital and EMS personnel • Financial concerns of smaller hospitals impact their willingness to participate in stroke center certification • Continuing education of EMT personnel • Resource allocation and state support • Data utilization and evaluation • Rural hospitals face unique obstacles • Unequal distribution and location of hospitals across the state
• What aspects of healthcare culture promote (or hinder) pre-hospital stroke care?	
• How do partners coordinate to develop the system of care? • Who or what agency conducts outreach to educate partners about changes to the stroke systems of care?	• Delegation of roles to coordinate care and provision of policy • Subcommittees are derived from conferences and meetings. They ultimately can steer the state protocols, EMS protocols, and educational processes • Clear communication channels • Hospital certification level development and expansion over the last decade has had an impact on the way stroke patient protocols are written/revised
• What other partners do you generally coordinate with while implementing new elements of the pre-hospital stroke systems of care?	• Partner roles include disseminating new SSOC policy intervention changes, functional roles within the SSOC, engaging closely with communities, and evaluating efforts and seamlessly sharing actionable information across partners
• How are procedures and protocols for your state's EMS system developed?	• Facilitators for advancing procedures and protocols include collaboration and sharing across elements of the SSOC, supportive leadership, uniformity, systematically tracking compliance, data-driven decision-making
• What challenges have you encountered while trying to adopt new practices in the field?	• Education and training as a consideration • Resource allocation as a consideration • Importance of buy-in • Level of experience and regional differences in care
• What types of practices/elements are more difficult to implement and why?	• Education and training as a consideration • Resource allocation as a consideration • Importance of buy-in • Level of experience and regional differences in care
• What are the jurisdictions/levels of your state's EMS system?	• EMS services managed by a combination of volunteers, city/county government, and private entities, resulting in gaps in EMS coverage • Private EMS providers set the geographic area they cover and provide services • No state mandates to provide ambulance services
• How are procedures and protocols for your state's EMS system developed?	• Protocol development involves EMS advisory committee or council • Protocols not implemented identically across the state • No state enforcement of protocols
• How do EMS providers across the state communicate with each other about pre-hospital stroke care?	• Regional EMS committees provide targeted feedback • Communication occurs at the agency level • Data quality improvement programs can serve as a foundation for communication across agencies
• Is there anyone supervising EMS between regions?	• Multiple partners involved in supervising/managing EMS systems at regional and state levels • Multiple points of contact for issues or complaints
• What factors are most helpful in facilitating implementation of changes to the stroke systems of care?	• Funding of work, personnel, and technologies • Evidence based approaches to EMS and hospital staff of evidence-based practices is crucial to secure buy-in and drive effective implementation. • Direct/close relationships and transparency • Workforce Development for EMS and elevating the importance of EMS professionals within a SSOC
• Is there a statewide or regional continuous quality improvement process that addresses weaknesses in the pre-hospital stroke system of care? If so, could you please describe it?	• Funding of work, personnel, and technologies • Evidence based approaches to EMS and hospital staff of evidence-based practices is crucial to secure buy-in and drive effective implementation. • Direct/close relationships and transparency • Workforce Development for EMS and elevating the importance of EMS professionals within a SSOC
• How do you measure performance?	• Use of validated assessment tools to transport/triage to appropriate facility • Reviewing/analyzing/evaluating data • Factors contributing to strength of program: clear communication, resources, education, relationships • Support for Stroke Center Certification
• Does your state have a system for stroke assessment?	• Use of validated assessment tools to transport/triage to appropriate facility • Reviewing/analyzing/evaluating data • Factors contributing to strength of program: clear communication, resources, education, relationships • Support for Stroke Center Certification
• What factors contribute to outcomes of your pre-hospital stroke system of care?	• Use of validated assessment tools to transport/triage to appropriate facility • Reviewing/analyzing/evaluating data • Factors contributing to strength of program: clear communication, resources, education, relationships • Support for Stroke Center Certification
• What are your state's plans for supporting ongoing development of the pre-hospital stroke systems of care?	• Funding best practices, statewide plans, and Coverdell Program • Support for Stroke Center Certification • Support for state (through training, conferences, data analysis and collection, and staffing) • Provider and community education • Importance of showing demonstrable improvement • Engaging professional organizations, tasks forces, and networks
• Are enough hospitals designated as stroke centers to improve EMS transport times?	• Proportion of hospitals that are certified stroke centers • Causes and mitigation of barriers to populations with geographically limited access to care • Causes and mitigation of barriers to populations with other types of limited access to care • Building practices and policies upon other time-critical care systems
• How many hospitals are currently designated as stroke centers across the state?	
• How does the stroke system of care fit in with other time-sensitive emergency response systems (such as trauma or STEMI/heart attack)?	
• How does your pre-hospital stroke system of care address access to care for difficult-to-reach populations?	

EMS, emergency medical service; SSOC, stroke system of care; STEMI, ST-segment elevated myocardial infarction.

### Challenge category 1: coverage of services, resources, facilities

A primary challenge noted by nine informants from five states was the physical distance to care for stroke patients in rural settings. Stroke center distribution did not always correlate with population density across a state and this issue was exacerbated for predominately rural states as hospital closures and staffing shortages occur due to remoteness and funding. Even with the presence of rural hospitals, informants noted that there were fewer specialists (e.g., interventional neurologists) in such locations whose presence on staff is required for stroke center certification, such as Primary Stroke Center (PSC), Comprehensive Stroke Center (CSC), Thrombectomy Stroke Center (TSC) or Acute Stroke Ready Hospital (ASRH). The combination of stroke center and staff scarcities translated to a lack of timely certified stroke care for rural populations. For example, one informant stated, “But we still have services around the state that still have difficulty attracting, you know, medical direction from board-certified ER [Emergency Room] physicians, just because they're so remote and they have such limited finances. So that can still be a barrier.” Informants also noted that EMS services were more prevalent in urban areas than in rural settings, often resulting in more time and longer distances for EMS personnel to reach stroke patients (13). Fewer individuals operating ambulances resulted in fewer ambulances out for services in the community, delays in reaching patients, and ultimately longer transport times to the hospital. Furthermore, informants noted that EMS jurisdictional organization contributed to gaps in access to care. Three states in our analysis reported relying on volunteers for some of their EMS services and others described the management of EMS across their state being a mixture of these volunteers, city/county governments, and private entities that set their service areas. The combination of fewer EMS personnel and ambulances and gaps in service areas may force patients to resort to the more time-consuming personal transport to the closest hospital rather than the transport most appropriate for their care.

### Strategies for addressing scarce resources, services, and facilities

Four strategies were proposed to address the challenges pertaining to physical distance to care. One key theme mentioned by informants was to explore ways of incorporating telemedicine. Two shared that remote consults by health care professionals in rural communities serve as a starting point to address prevention for at-risk stroke patients. Two other informants suggested incorporating real-time stroke telemedicine on ambulances, which allows EMS professionals and stroke centers to communicate enroute to the hospital (14). Additional benefits of this strategy include the ability to capture unique stroke metrics including the geographic location of stroke centers, the location of stroke patients when EMS was called, where the ambulance picked up the patient, and which hospital they were transported to. For instance, one informant stated, “And really from a telemedicine side …. you know we have parts in the state where there's no Internet access. You know, they just can't get a good Internet signal, and so telemedicine even becomes a challenge. And so if there was more money we could certainly – you know but it's that access to care. It truly is and even if we could get telemedicine into some of these parts of the state, we would be changing it vastly but it's just hard in certain sections.” The use of software to track similar metrics was noted as one way to compare and improve stroke metrics between states (13). Additional strategies noted by two informants included utilizing a state-wide 24/7 communications center staffed by trained medics who assess patient needs and advise the attending medic on the closest appropriate facility, as well as dispatching volunteer fire departments in remote areas to provide initial stroke assessments and life preserving procedures until paramedics can arrive.

Identified strategies to support stroke center certification included informal and formal methods, ranging from promoting the public's awareness of availability to regulation. One informant who identified as a practitioner in the field, shared “Well, I do think that the stroke certification is useful to the EMS community because that designation can tell them immediately what kind of capacity that rural hospital might have, and so there's great value to me in the stroke designation. I think it would be helpful if every hospital had some sort of designation as it relates to strokes so that the provider, the EMS practitioners could make the decision about closest, more appropriate facility for transport to.” Keeping EMS personnel abreast of what levels of stroke care are available in surrounding hospitals allow them to better choose transportation routes based on the condition and needs of the patient. Three states described their strategies to advance hospital stroke center certification through regulation, competition, and encouragement. An informant from a state health department noted that implementing state regulations prioritizing stroke certification has encouraged hospitals to compete to become certified as well as to increase the level of certification. Another informant described instances where legislative mandates become important in cases where a single hospital dominates a given geographic area and lacks market incentive to become certified.

### Challenge category 2: standardized statewide SSOC protocols and tools

Creating standards for stroke protocols, including the consistent use of severity and transport/triage tools as well as comprehensive and widespread EMS training, was identified by informants as important for initial support of statewide SSOC policies. However, informants expressed challenges with enforcing evidence-based practices within a SSOC due to the lack of mandatory or recommended practices by the state or an overseeing body of the state's SSOC. A related challenge to the content of SSOC protocols was the absence of comprehensive and widespread emergency response training. EMS responders across a state may have varying degrees of experience and challenges arise with ensuring emergency response personnel are kept up to date with the rapidly evolving changes in stroke care. Two informants representing different states mentioned that, although it would be ideal, state-directed trainings often are not feasible for geographically dispersed EMS professionals to attend. In addition, informants expressed that disagreement occurs between providers in the pre-hospital setting about adopting new or updated stroke training curricula and protocols. One informant representing a hospital association said that retiring older practices and implementing emerging science-backed practices can meet resistance, especially if EMS providers aren't shown years of research that backs the new protocol: “If you took a class from [university], they have a very extensive advanced stroke life support program that teaches a specific type of quantitative scoring on the MENS scale whereas we in [state] use RACE, and to try to get people to adopt race after they've been using MENS is a challenge. Let's face it, there is resistance to change out there and nobody likes change as much, and EMS and the fire service knows we're resistant to change… You know, EMS providers need to be shown the data… we're in the midst of gathering the data right now and until five, 10 years of research can show that on a pre-hospital level, this is what's most beneficial, there's gonna be that resistance.” Such changes are compounded for volunteer-based EMS agencies located in rural areas who equally need to maintain high education standards but often require more continuous education on stroke protocols due to frequent turnover of volunteer staff.

### Statewide SSOC protocol and tool strategies

While informants expressed the need to build a statewide SSOC, they noted to facilitate implementation, the policy development process needed to represent perspectives of those implementing practice across the SSOC. For example, informants stated that practice-based evidence needed to be implemented into state rules and regulations at a rapid pace. Informants stated that feedback from a variety of practitioners across the stroke system of care network to state decision-makers throughout the policy development process allows for improved statewide consensus and sets expectations when relating to the content of the proposed SSOC legislation. Identified perspectives included representatives from councils, committees, and associations that make recommendations for pre and in-hospital stroke care. Additionally, informants noted the importance of including hospitals that serve rural areas and have varying certification levels (i.e., ASRH, PSC, TSC, CSC) as part of this process.

Informants from three states shared that their states made efforts to streamline statewide use of validated stroke severity and assessment tools in order to correctly identify stroke patients and transport them to the appropriate facility. They emphasized that to ensure compliance, these stroke assessment tools and transport protocols, which are typically outlined in statewide protocols, should be mandated. A representative from EMS stated that having these protocols standardized by either the state or regional stroke task force was important to ensuring expediency in the patient care decisions made by trained EMS professionals: “there's a lot of dispute among the hospitals whether or not the training of the EMS has been uniform with regard to recognizing the severity. And there's been recent changes in the protocols that take effect in January [of 2020] which also concern a number of the hospitals in terms of transport. I'm not a doctor nor do I try and make a clinical determination as to the science. However, unfortunately here in [state] our protocols are dictated in large part by an ambulance advisory committee. And while we have a stroke task force, it's advisory as well in nature, and its input – it's not structured.”

While some informants were supportive of more uniformity, they also recognized the need for flexibility in the implementation of these protocols due to regional differences and composition. According to informants, a region-wide or statewide system would not be supported in some cases. For example, one state allocated medical directors of individual service providers with the authority to establish SSOC protocols. Another state's emergency response network has a statewide protocol; however, not all regions or EMS or hospital entities are mandated to follow these state-developed protocols.

Informants shared the importance of providing accessible, evidence-based, and needs-based training curriculum in alignment with the state's standards. Specific recommendations from the informants for EMS training included providing virtual vs. in-person modalities to ensure accessibility to training for remote staff. Informants from two states expressed the need for video recorded trainings that allow personnel to conveniently access this resource via the EMS website and earn credit for the training. Both informants stated that funding for such online training management systems was a barrier. Additionally, tailoring education to local rather than just statewide needs was important. Informants from two states mentioned that holding state facilitated meetings was preferred if state-directed training was not feasible. In this model, each state's maximum involvement included setting baselines and statewide standards for pre-hospital care education and having annual meetings covering a broad range of topics. Lastly, states' use of professional organizations and programs as an additional resource for ongoing training and education was recognized as an efficient strategy to provide timely EMS training across the state.

### Challenge category 3: SSOC quality and performance improvement

Informants expressed that the absence or lack of participation in a stroke data registry hindered continuous feedback loops between EMS and hospitals and therefore the ability to improve quality of care. These challenges were noted as more acutely existing in under-resourced EMS and hospitals serving smaller populations, such as ASRHs. A couple of challenges existed with stroke registry data collection. First, data were not being collected, or are incomplete because data were not flowing between pre-, in-, and post- hospital settings and second, if data were being collected, there was no one to track and analyze the data, such as patient outcomes like death and disability or readmissions to the emergency department.

### SSOC quality and performance improvement strategies

To address stroke patient data challenges, informants suggested using data-driven programs and organizations such as the Paul Coverdell National Acute Stroke Program (15) and stroke professional organizations as resources for EMS professionals and stroke coordinators. These resources can provide training, data collection, and quality assessments, further enhancing standard practices across the state (16).

An informant shared that they, “participate along with several other organizations in the Coverdell Stroke Registry's pilot programs for EMS and Coverdell has been very beneficial in helping to drive some standardization within our services areas.” A state stroke taskforce member and EMS representative on the task force from the same state agreed to the benefits of having an in-house epidemiologist to facilitate data analysis across the state and identify gaps in data collection. Their state conducts an annual stroke taskforce review of protocols and stroke outcomes to improve the quality of their system of care across the state. Financial incentives are another way to promote the use of stroke registries as demonstrated in one state which reimbursed hospitals for the costs associated with participation in the American Heart Association's Get with the Guidelines Registry, a nation-wide intiative (17).

Informants shared that communication and work culture strategies can improve aspects of SSOC quality and performance. One informant shared an example of how they used communication to strengthen understanding between the two entities by maintaining close contact with both hospital and EMS personnel. This process included trying to understand patterns behind the stroke patient data and EMS professionals' perspectives from the field when entering stroke patient data. This communication, sometimes informal, can be critical to understanding patterns behind stroke patient data and improving processes for data collection and entry. Additionally, at least two informants shared that establishing trust and bridging differences can aid in ease of communication and maintain collaboration, respectively. Similarly, positive reinforcement through transparent communication between hospitals and EMS can help improve care delivery. An example provided was inviting EMS professionals to be a part of stroke conferences and alliances and acknowledging their contributions, further driving improvements in care delivery.

## Discussion

A recent analysis concluded that many states have SSOC laws supported by evidence; however, there are gaps in how state laws integrate this evidence into a SSOC (5). The Institute of Medicine recommends that state and local governments work with their public health agencies to review existing public health laws to ensure that the challenges to population health are addressed (18). Further, the American Stroke Association recognizes the need for a national consensus approach to acute stroke care that considers differences in regional plans in urban, suburban, and rural environments (19, 20). The results from our analysis identified several strategies for effectively implementing pre-hospital SSOC policies that may mitigate geographic disparities in access to care.

To address unequal coverage of services, resources, and facilities in rural areas, state support for telemedicine and improved access to certified stroke centers were identified as important strategies for improving access to time-sensitive, high-quality stroke care. Telemedicine remains an emerging area for varying health care settings and conditions, including stroke, and early evidence indicates that the potential for thoughtfully implemented technology may improve stroke outcomes (21). The Brain Attack Coalition proposed certification of ASRHs in 2013 to encourage rural or smaller facilities to diagnose and initiate stroke treatment using telemedicine (22). A challenge with implementing telemedicine in rural communities includes disproportionally less internet broadband access; according to a recent survey, less than half of census tracts representing Rural-Urban Commuting Areas met the Healthy People 2020 objective of 83.2% (23). Additionally, many rural areas are considered health professional shortage areas and have seen hospital closures over the last decade (24, 25).

Study findings suggest that statewide regulatory approaches that enhance standardization have the potential to improve effective coordination within regions and statewide. A diverse range of SSOC perspectives and involvement in the development, implementation, and enforcement of stroke protocols can help inform the establishment of consistent protocols and tools for transport, triage, and transfer of stroke patients. Successful implementation can be achieved through both informal and formal engagement of key implementers in statewide taskforces and coalitions.

Needs-based and flexible approaches for training stroke professionals can help with maintaining quality control and protocol adherence, particularly in remote areas of a state. Adaptation of the educational curriculum and practices of other time-critical systems can strengthen SSOCs. Further, data quality assessments and collaboration among partners can enhance performance and improve quality of care. These findings are also supported by a recent impact and case study analysis (26). To our knowledge, no one has published a similar qualitative study examining implementation strategies to address rural disparities in access to care for stroke patients. The results from our analysis revealed several potential approaches public health practitioners could take to enhance the effective implementation of SSOC policies. This work will involve working collaboratively with partners across SSOC on a regular basis to help diminish inequity in treatment of stroke, improve health outcomes for stroke patients, and save lives.

## Limitations

There are important limitations to our study, including sample size and potential for bias. First, while there were efforts to ensure representation across states and interviewee perspectives through delegate categories, there is a potential for selection bias of informants through initial contact of EMS/state medical directors. Two delegate categories with less representation than the other six categories were in the “disability” and “data” categories. Second, because some informants participated in small group interviews, there is potential for groupthink tendencies. Third, due to stipulations under the Paperwork Reduction Act (PRA), the Office of Management and Budget prohibited CDC staff from direct receipt of informant personally identifiable information, thereby preventing us from breaking down results by specific informant titles. Further, also partly due to PRA, we were limited in our capacity to conduct a 50-state analysis with a more representative sample of informants, limiting applicability of findings. However, we attempted to create variation in our study sample by selecting states that had different policy compositions (i.e., legislative, regulatory, or both), states that did not meet our specific inclusion criterion (i.e., do not have a law authorizing a SSOC with at least three levels of stroke centers) are not represented in this study and present a major limitation for representativeness of all states.

## Conclusion

Many challenges exist to improve coordination and delivery of care to stroke patients in rural communities. However, there are strategies that can be incorporated at the state level within SSOC to advance effective, evidence-based policy implementation. The mitigation strategies from this study were identified by practitioners on the ground who encounter and attempt to resolve these issues daily. Utilizing these strategies could help state and local practitioners address challenges concerning access to timely stroke care for all patient populations.

## Data Availability

The original contributions presented in the study are included in the article**/Supplementary Material**, further inquiries can be directed to the corresponding author: aysharasool@outlook.com.

## Footnotes

^1^Policy can be defined as “a law, regulation, procedure, administrative action, incentive, or voluntary practice of governments and other institutions (9)”.

^2^Home rule status refers to the authority delegated to local governments to enact their own laws and policies to address issues of local concern.
