# Supplementary material for: Policy implementation strategies to address rural disparities in access to care for stroke patients
Source: Front Health Serv. 2023 Nov 30;3:1280250. doi: 10.3389/frhs.2023.1280250 (PMC10733855; doi:10.3389/frhs.2023.1280250)
Supplement: Supplementary file 1 [file Table1.docx]

**Supplemental Digital Content Table S1** Interview Guide

Form Approved

OMB No. 0920-0879

Expiration Date 01/31/2021

**Instructions**

Welcome! This data collection is meant for state, local, tribal, or territorial health department staff and their delegates. It aims to explore the nature of your state’s pre-hospital stroke interventions and systems of care, including the implementation process and any impacts or outcomes you have experienced as a result.

Your feedback is important to us and will help us develop technical assistance material for state grantees and emergency medical service system (EMSS) coordinators that will be disseminated via conference presentations, webinars, fact sheets and implementation guides. Findings may also be published in academic journals.

On this call today is [interviewer] from ASTHO’s Research and Evaluation team who will be conducting the interview, as well as [note taker], who will be taking notes. This work is being funded by CDC.

**Completing the interview is voluntary and takes approximately [90] minutes.** Personally identifiable information such as your name and position will not be collected. However, your role and state may be published. There are no known risks or direct benefits to you from participating or choosing not to participate, but your answers will help CDC improve technical assistance related to pre-hospital stroke interventions and systems of care.

I am going to ask you a series of questions about pre-hospital stroke systems of care in your state. Keep in mind there are no right or wrong answers, and we are grateful for any information you can provide. If there are any questions to which you do not know the answer or any questions that you do simply do not want to answer, just say pass and we will move on. You are welcome to stop the interview at any time.

If you agree, we will record this interview and develop a complete transcript that will be used for qualitative data analysis. The recording will be used internally and will be destroyed after we’ve completed our analysis. Would you be comfortable with that?

• If yes: Great. Thank you so much. If you could hold for one moment, we’ll start the recording.

• If no: Ok, no problem.

Do you have any questions for me before we begin? If not, let’s get started.

**Role summary**

To begin, we are going to be talking about the actions that have been undertaken at the state or regional levels to improve pre-hospital stroke care practices in your state. Examples of the kinds of things we’re talking about include stroke pre-notification, triage, transport, and transfer of patients to the most appropriate stroke facility. These particular examples may or may not be applicable to your state.

First, we’d like to identify your role in these kinds of interventions. Would you define yourself as:

- - **Developer:** Someone who works at higher levels to develop new stroke care practices or shape the pre-hospital stroke system of care
  - **Implementer:** Someone who actually works with and implements pre-hospital stroke care practices
  - **Both**

**I. Development**

1. Please describe the general rollout process for changes in the pre-hospital stroke system of care.

- Are there regional differences in the rollout?

1. What are the greatest obstacles for pre-hospital stroke treatment in your state?
2. What is the easiest part of the development process?
3. Who are the stakeholders involved with helping to develop the pre-hospital stroke systems of care? Please provide agencies and roles, if possible.
4. How do stakeholders coordinate to develop the system of care?
5. Who or what agency conducts outreach to educate stakeholders about changes to the stroke systems of care?
6. How many hospitals are currently designated as stroke centers across the state?
   - Has this changed over time?
   - Are enough hospitals designated as stroke centers to improve EMS transport times?
7. What aspects of healthcare culture promote (or hinder) pre-hospital stroke care?
8. How does the stroke system of care fit in with other time-sensitive emergency response systems (such as trauma or STEMI/heart attack)?

**II. Implementation**

1. What other stakeholders do you generally coordinate with while implementing new elements of the pre-hospital stroke systems of care?
2. What challenges have you encountered while trying to adopt new practices in the field?
3. What types of practices/elements are more difficult to implement and why?
4. Can you think of some form of support that your state could provide to make a positive impact on pre-hospital stroke care?
   - What kinds of support do you already receive?

**III. EMS System**

1. What are the jurisdictions/levels of your state’s EMS system?
2. How are procedures and protocols for your state’s EMS system developed?
   - How are these communicated and enforced?
3. How do EMS providers across the state communicate with each other about pre-hospital stroke care?
4. Is there anyone supervising EMS between regions?

I**V. Program improvement, outcomes and sustainability**

Program improvement

1. What factors are most helpful in facilitating implementation of changes to the stroke systems of care?
2. Does your state have a system for stroke assessment?
   - For stroke triage?
   - For timely transportation to the most appropriate level of care?
3. How does your pre-hospital stroke system of care address access to care for difficult-to-reach populations?
4. How do you measure performance?
   - Are performance measures for the stroke system of care state actions collected at local, state, and/or regional levels?
5. Is there a statewide or regional continuous quality improvement process that addresses weaknesses in the pre-hospital stroke system of care? If so, could you please describe it?
   - How is quality improvement information shared between state and clinical settings?
   - Do you have specific reporting requirements?
6. How is the pre-hospital stroke system of care enforced?

Outcomes

1. Does your state assess the outcomes of the pre-hospital stroke system of care?
   - Please describe your assessment process including types of data you are collecting.
2. Could you describe any improvements you’ve seen in pre-hospital stroke patient care due to the practices implemented by the state?
3. What factors contribute to outcomes of your pre-hospital stroke system of care?
4. Are there gaps or areas for improvement needed for your pre-hospital stroke care system?

Sustainability

1. What are your state’s plans for supporting ongoing development of the prehospital stroke systems of care?
2. Given your experience, is there anything unique or innovative about your state’s experience that you think other states would be interested to know?

-END-
