# Supplementary material for: Policy implementation strategies to address rural disparities in access to care for stroke patients
Source: Front Health Serv. 2023 Nov 30;3:1280250. doi: 10.3389/frhs.2023.1280250 (PMC10733855; doi:10.3389/frhs.2023.1280250)
Supplement: Supplementary file 2 [file Table2.docx]

**Supplemental Digital Content Table S2** Challenges, Related Quotes and Mitigation Strategies

| **Challenge** | **Related Quote** | **Mitigation Strategy** |
| --- | --- | --- |
| Coverage of EMS services are not comprehensive and do not cover the entire county or state | - “…Some of these ambulance services are covering jurisdictions that come close to 3,000, 4,000 square miles of territory that they respond to. So we're spread very thin.” - “The other thing to understand is that [in] [state], like most other states and territories in the union, there is no statutory mandate that anyone or any political subdivision provide ambulance services.” - “The other clarifying point to put on this is to say: we do not specify to any ambulance service, be they air or ground, "This is your territory and this is where you cover." So roughly across the state they follow their county lines. But, again, that's not always the case.” | - Enabling state support for the integration of tele health |
| Expanded acute stroke care to all areas of the state due to outdated/non-existent technology | - “And really from a telemedicine side …. you know we have parts in the state where there’s no Internet access. You know, they just can’t get a good Internet signal, and so telemedicine even becomes a challenge. And so if there was more money we could certainly – you know but it’s that access to care. It truly is and even if we could get telemedicine into some of these parts of the state, we would be changing it vastly but it’s just hard in certain sections.” - “And then we [are] building out a system of care for stroke. As I mentioned, our hospital serves as a hub for a telestroke system that currently involves four other facilities. And hopefully there'll be as many as nine or ten facilities by the end of the year. And each of those facilities looks to us for direction on the development of their pre-hospital policy. So we're having influence over things like pre-hospital notification, care for the stroke patient in the field, and increasingly getting involved in real-time decisions about transportation of the patient to the facility they should go to. So the mechanism for interaction is the telestroke system itself.” - “And they take that information and produce a geographic mapping, among lots of other things. And so they're able to show you maps of where the hospitals are. They have syndromic analysis available so they can look at – depending on your definition, they can look at stroke patients. And they can show you how many stroke patients are within a certain radius of different hospitals within your system.” | - Enabling state support for the integration of telehealth |
| Unequal personnel and resource allocation in remote areas | - **“**But we still have services around the state that still have difficulty attracting, you know, medical direction from board-certified ER [Emergency Room] physicians, just because they're so remote and they have such limited finances. So that can still be a barrier.” - “I would just say that especially for rural states, it’s really important to get this right. But it’s just become increasingly challenging with more and more providers being in an urban settings, and more and more rural hospital closing. This problem doesn’t get easier, it just gets harder.” - “We find that there are some of our rural facilities, and I’m sure it’s not just [state], but some rural hospitals are limited in their resources. And when you think about the amount of rural hospitals that have closed over just in the past few years here, and those that are still financially unstable throughout the country, we have about 46 percent of our hospitals here that are financially unstable, having enough resources to facilitate these type of processes, whether it’s stroke or anything else, can be extremely challenging. . . . That’s one of, I think, one of the significant barriers is that it’s ensuring that they have the resources they need to make sure that we’re – that they’re successful…. But really for us we have done such a great job of being able to work with our hospitals that it’s really we don’t have huge barriers. I think the infrastructure for hospitals they have their own barriers, like I said, with lack of resources.” - “Was hard at one-time, to define policies of who gets what. And so, I think when you have three groups working together in the same arena, hospitals, providers and EMS, there is a concern with allocated resources whose getting what. Is somebody getting - their disproportionate share? Is an area I think that they deserve more than the other. So, I think that’s always a fair and natural intention.”   Quotes supportive of strategies:   - “Well, I do think that the stroke certification is useful to the EMS community because that designation can tell them immediately what kind of capacity that rural hospital might have, and so there’s great value to me in the stroke designation. I think it would be helpful if every hospital had some sort of designation as it relates to strokes so that the provider, the EMS practitioners could make the decision about closest, more appropriate facility for transport to.” - “….Something that we see is that, we have varying degrees of skill level in our ambulance services – from first responders through critical care, certified care medics. So we aren't seeing, in some services, the identification of some of the large vessel occlusions. I think that they are great at identifying strokes. But in the Southeast region, there are three certified stroke facilities within this area. And some of the transport times that we see are very long – hour, hour-and-a-half. So we are seeing a lot of patients come in by air to us, or depending on the patient's condition, they are stopping at outlying facilities and then being transferred to us as driven to by the station.” - “We implemented, in 2015, having our EMS here in [omitted] County call our neurologist directly versus calling the ER and then the ER calls the neurologist. So that gives the EMS that direct communication with the neurologist. The neurologist can say, ‘Yes, activate a code stroke,’ or, ‘No, I don't think that's a stroke.’ So having that real-time conversation and real-time feedback gives buy-in and just helps them be part of a team.” | - Encouraging EMS to be an integral part of the interdisciplinary stroke team - Greater availability of certified stroke centers |
| Statewide uniformity among use of validated stroke severity scales and transport methods | - “there's a lot of dispute among the hospitals whether or not the training of the EMS has been uniform with regard to recognizing the severity. And there's been recent changes in the protocols that take effect in January [of 2020] which also concern a number of the hospitals in terms of transport. I'm not a doctor nor do I try and make a clinical determination as to the science. However, unfortunately here in [state] our protocols are dictated in large part by an ambulance advisory committee. And while we have a stroke task force, it's advisory as well in nature, and its input – it's not structured.” - “…as far as enforcement goes…. that's a difficult thing to be in a position for, especially with a volunteer agency. The resources that it requires to create protocols is enormous. The knowledge and the amount of research that goes into that. So there's not really any enforcement. The state is starting to improve enforcement for documentation on run reports. But I don't know, again, that there's any enforcement done on protocol side.” - An example of how one state requires specific practices: “But when we push out something, sometimes it is a requirement. So, while we do value our stakeholder input, we do kind of have a little bit different push there for them to maintain licensure they have to do these certain things. So, for example, for the EMS agencies, we tell them that they have to have two different protocols. They have at least two for stroke. One of them is a stroke triage tool and one's a severity tool. So even though every single EMS agency did not necessarily input into the process, every single EMS agency will participate in the process because they have to do that.” - “And there is - there is always unexpected challenges that pop up. For example, when are - when we instituted the use of the LAMS Score, the - so the Comprehensive Stroke Center had a they have a policy was that, they would get somebody - somebody was presented with stroke symptoms they take them right to the CAT Scan. So, they didn’t do a severity score in the hospital. So, EMS was reporting the severity score - the LAMS score when they arrived. And some nurses would say, ‘Oh, we don’t care, we don’t use that.’ What they meant was, they weren’t going to take the time to do another one. They were going right to the CT Scanner no matter what. So, then that got translated back to some of the EMS practitioners that the LAMS Score wasn’t helpful. So, again, that was an unexpected challenge were able to overcome it, but again so we had to do a little more outreach and work with that.” - “I mean I think with any change you run into folks that are – the line is always that’s the way we’ve always done it and so I think sometimes breaking down some of those – the old way of doing things can be challenging” - “If you took a class from [university], they have a very extensive advanced stroke life support program that teaches a specific type of quantitative scoring on the MENS scale whereas we in [state] use RACE, and to try to get people to adopt race after they’ve been using MENS is a challenge. Let’s face it, there is resistance to change out there and nobody likes change as much, and EMS and the fire service knows we’re resistant to change… You know, EMS providers need to be shown the data… we’re in the midst of gathering the data right now and until five, 10 years of research can show that on a pre-hospital level, this is what’s most beneficial, there’s gonna be that resistance.”   Quotes Supportive of strategy:   - “some kind of state-driven protocols where they're uniform….things are done vastly different from service to service….some sort of state guideline, I think it would be helpful….it should be a little more uniform…require overcoming the diversities of both geographic and logistical differences between the services… uniformity to the stroke system from the state level” - “consistency…. guidelines, you might say, to make sure that everyone is consistent with what out-of-state level is going on.”   Quotes Supportive of state-mandated protocols:   - “So I'm going to say, probably protocol adoption. You know, we can create model protocols. We do not have a statewide protocol in [state] like some states do. Some say that's good, and some say it isn't. [Laughs] But in one respect, protocol adoption, having the engaged medical director at the local ambulance service is not always there. And when you don't, you got a director that's trying to effect change and the medical director may or may not be willing to go along with that based on their own knowledge and understanding. So, I mean, that can be a challenge. We try to embrace those folks and communicate and provide information to them to help change that. And it meets with various levels of success depending on the individuals.”   Quotes Supportive of flexibility in implementation of stroke protocols:   - “So from a state-wide standpoint, we make our recommendation but we leave it up to local regional locations to kind of roll out or implemented as needed, based on current challenges and dynamics, geography, resources that are available.” - “So protocols can differ some or have differences depending on what their resources are in the proximity to their service areas, but outside of that, it's pretty much streamlined, I believe, overall.” | - Advocacy, development & enforcement of mandatory statewide protocols - Consistently use the same severity scale between EMS and receiving hospitals |
| Absence of statewide-managed stroke system of care network/ state involvement | - "This is important and we need the funding and we need the infrastructure in place in order to do that." Because to me it's not acceptable for [state] to just say, "Eh, there are no solutions to be had." There are solutions to be had. It's just: we need other policy makers and decision makers to hear what the difficulty is in rural health care in a very rural state. And anything that can be done to influence that from the top down so that [name] and [name] who are actually caring for people, so that they have what they need, that's my pitch, in saying that's really what we need in [state].” - “I think inclusion of the prehospital community in the development of any new protocols prior to adoption is helpful and valuable.” - “We're trying to create a statewide consensus on this stuff.  If you have that, when you go to getting the regulations finalized and approved, you're less likely to run up against obstacles.” - “But I really think the lacking part here is the state involvement in creating a stroke system. Because, as we already mentioned, part of the problem with the protocols is that it's done by each individual agency. The stroke system is really done by each individual locale. So us with our hospital or with any hospital that's willing to work with us. It's not a statewide system.” - “So, both our regular attendees at The Stroke Taskforce as are the stroke care coordinators network, and everybody I think that sits in those positions is passionate about what they do when they attend the meetings in order to take their feedback and push it back out to EMS in far as what they’re seeing as we continue to tweak and continue to learn, and create a culture of continuous quality improvement and not punishment.” | - Engaging with represented stroke coalitions, stroke champions, medical directors to bridge relationships between EMS-Hospitals-State. |
| Absence of stroke registry or lack of participation in stroke registry | - “Yeah, I think one of the criticisms of our program, and certainly an area of improvement is how do we all - in all of those areas have access to the same data, and move it freely between those three groups, hospitals, providers, and EMS. I think that’s one of our real Achilles Heels.” - “...we participate along with several other organizations in the Coverdell Stroke Registry's pilot programs for EMS and Coverdell has been very beneficial in helping to drive some standardization within our services areas” - “But I think that, that and the data collection and analysis and the - any feedback that we can, you know, that the nurses and the hospital division can provide to the EMS providers is super valuable.” - “What's still missing is: none of the hospitals that we're working with currently are participating in any data submission to a stroke registry. And so we don't have full data sets on performance of the facilities that we're working with. And for me that's one of the biggest missing elements.” - “But I think that, that and the data collection and analysis and the - any feedback that we can, you know, that the nurses and the hospital division can provide to the EMS providers is super valuable.”   Quotes supporting strategy:   - “We participate along with several other organizations in the Coverdell Stroke Registry's pilot programs for EMS and Coverdell has been very beneficial in helping to drive some standardization within our services areas.” - “So, I guess from the standpoint of what you’re saying, I think the support of an epidemiologist for data analysis and data review has been helpful. And I would encourage other states to support their stroke programs in that manner.” “having epidemiologist are - it’s invaluable to analyze the data and identify areas where we can do better as far as data collection. But also, helps us understand what’s going on across the state.” - “So I think that that was the starting point for the state office as far as pushing this out to ambulance services to say, ‘Hey, look, your data is important, we're looking at it. This is where you stand in regard to some of the other agencies in the state.” | Efforts through incentivization, critical feedback, and data driven programs |
| Absence of emergency response training for treating stroke patients | - “I think education is always a challenge. Making sure that everybody receives the information. [There are some individuals] who may drag their feet or not agree with changes that were made. So I think that's always a challenge.” - “Teaching the nuances [is] a challenge [that] again give to disparity and education and training and level of practice and how active all of [our] EMTs are.” - “one of the biggest challenges is that in the field of stroke, the advances are so fast that we have trouble keeping up with the educational needs for our state – and keeping up with the protocols, for example. I mean, it's great that our field is advancing so quickly.  We just sometimes struggle to keep up with it.”   Quotes supporting strategy   - “And then education. When we find a need, specifically a need that's occurring at the present time – not just a statewide need but a local need – we will provide training on that need. So we don't just provide training to get continuing education hours; we provide it based on needs. I'd say those are our strengths.” - “I think education is an absolute must. And that is probably one of the number-one things that would affect the implementation of any changes to the stroke system of care.” - “Video training and we would record it and put it on the EMS website and whenever the personnel at any point in time, they have time to go up on the website and go over the presentation, it will provide them credit.” - “an online learning management system so that we could publish trainings on all of this and have it free, and have it where, especially in rural [state]” - “Every time I'm presenting to a group of EMS personnel, we talk about our systems of care, and so we emphasize all of them like trauma, stroke, cardiac, the main three that we deal with, but when we make one system better, we make the other systems better.” - “And none of that comes at the direction of any state agency… there's a fairly limited set of opportunities for education at the state level, which only happens during a certain time of the year and hasn't really meshed with the schedules of some of the providers who would otherwise like to be involved in that.” - “a learning management system that would help our end user to be able to track when they utilize our educational offerings … but that’s a funding problem” - “...We’re looking to model off of the regionalization of how trauma regionalized and how STEMI care or cardiac care is regionalized so that it can – those processes can be improved upon from – on a regional basis, not state-wide, but at the same time, affect state-wide management of these conditions.” | - Leveraging and learning from other time-critical care systems - Flexible, evidence-based, and needs-based education to encourage buy-in of practices - Statewide standards for education influenced through on the ground personnel |
